# Pelvic fractures in severely injured children: Results from the TraumaRegister DGU

**DOI:** 10.1097/MD.0000000000011955

**Published:** 2018-08-21

**Authors:** Jörn Zwingmann, Rolf Lefering, Dirk Maier, Lisa Hohloch, Helge Eberbach, Mirjam Neumann, Peter. C. Strohm, Norbert P. Südkamp, Thorsten Hammer

**Affiliations:** a Department of Orthopedic and Trauma Surgery, University of Freiburg Medical Center, Hugstetter Strasse 55, Freiburg; b Institute for Research in Operative Medicine (IFOM), University of Witten/Herdecke, Cologne; c Clinic for Orthopaedics and Trauma Surgery, Klinikum Bamberg, Germany.

**Keywords:** children, pediatric pelvic fracture, pediatric pelvic fractures, pelvic fracture, TraumaRegister DGU

## Abstract

Injuries in the pelvic region in children and adolescents are very rare and often associated with a high energy trauma. Aim of this prospective multicenter study was, by analyzing the data from the TraumaRegister Deutsche Gesellschaft für Unfallchirurgie (TR-DGU), to evaluate any correlation between the severity of pelvic fractures and resulting mortality in different age groups.

These study findings are based on a large pool of data retrieved from the prospectively-setup pelvic trauma registry established by the German Trauma Society (DGU) and the German Section of the Association for Osteosynthesis/Association for the Study of Internal Fixation (AO/ASIF) International in 1991. The registry provides data on all patients suffering pelvic fractures within a 14-year time frame at any 1 of the 23 level 1 trauma centers contributing to the registry. The analysis covers 4 age groups ranging from 0 to 17 years, covering different factors regarding pelvic fractures and their treatment.

We identified a total of 9684 patients including 1433 pelvic fractures in children aged ≤17 years. Those patients were divided into 4 subgroups according to the patients’ age (groups A–D) and according to the fracture severity (group 1 =  Abbreviated Injury Scale (AIS) score pelvis ≤2, and group 2 = AIS pelvis ≥3). The mortality in group 1 was 8.8% with a RISC (Revised Injury Severity Score) II of 8.6%, standard mortality rate (SMR) of 1.02 and 7.2% in group 2 with an RISC II of 9.9% (SMR 0.73). In pelvic factures of Type A (Tile classification of pelvic fractures), an SMR of 0.76 was recorded, in Type B fractures the SMR was 0.65, and in Type C fractures 0.79. Severe pelvic injuries (AIS pelvis ≥2) were associated with a higher rate of whole body computer tomograph (CT) scans (1–5 years: 80%, 6–10 years 81.8%, 11–14 years 84.7%, and 15–17 years 85.6%). The rate of pelvic surgery rose with the pelvic injury's severity (AIS 2: 7.6%, AIS 3: 35%, AIS 4: 65.6%, AIS 5 61.5%). We observed higher rates of preclinical and initial clinical hypotension defined as Riva-Rocci (RR) <90 mmHG) as well as of preclinical fluid application in all age groups. The presence of a pelvic injury was associated with a higher rate of severe abdominal injuries with an AIS of ≥3 (25.1% vs. 14.6%) and of severe thorax injuries with an AIS≥3 (43.6% vs. 28.6%).

We have been able to analyze an enormous number of pelvic fractures in children and adolescents including different age groups by relying on data from the TR-DGU. Mortality seems to be associated with the severity of the pelvic injury, but is lower than the RISC II score's prognosis.

## 1 Introduction

Trauma and its associated injuries are the commonest cause of death in children.^[1]^ Pelvic trauma and fractures in children and adolescents are rare with an incidence between 2.4 and 7.5%^[2,3]^ and the mortality is significant with a reported range of 1.4% to 25%.^[4–6]^

Most patients with a pelvic fracture are therefore multi-traumatized patients with injuries to the head, chest, abdomen, and extremities.^[6]^ High-energy traumas are the leading cause for these injuries.^[2]^ The morbidity and mortality associated with them are usually higher in comparison to other types of orthopedic trauma.^[7]^ Keshishyan et al identified in a postmortem study of trauma patients a high rate of pelvic fracture-related deaths and a high incidence of pelvic fractures.^[8]^ Because of multiple life-threatening injuries caused by high energy trauma,^[9]^ the child's initial treatment is usually less focused on pelvic injury. Moreover, cardiopulmonary resuscitation (CPR) in children after severe trauma seems to result in a better outcome than in adults, and appears to be more justified than current guidelines would imply. Resuscitation in the emergency room (ER) is even associated with a better neurological outcome compared to resuscitation in a preclinical context or in both the preclinical phase and ER.^[10,11]^

Children's pelvic bones are less brittle, covered with thick periosteum, more elastic, and more cartilaginous than an adult's. Moreover, the bony matrix is flexible, the ligaments are relatively stronger, and growth centers are still present which, together with the sacroiliac joints and pubic symphysis, enable a significant shock absorption capacity.^[12]^ The fragile points in child's pelvis are the triradiate cartilage and the sacroiliac joint. This elasticity primarily enables plastic deformation when the pelvic bone absorbs an impact.^[13]^ This plastic deformation leads to restoration of the pelvic anatomy, although not necessarily to the pre-injury level. Such elasticity means that the intrapelvic viscera are insufficiently protected, and intrapelvic organs can suffer injury even without obvious pelvic fractures or dislocations.^[14]^ Isolated pubic rami fractures or iliac wing fractures are the most frequently associated fractures in the pelvic region of children and adolescents.^[6,9,15]^ A complete disruption of the anterior and posterior pelvis or a complex pelvic injury is associated with a high risk for morbidity and mortality.^[16]^

Fatal hemorrhage as noted in adult patients with pelvic fractures is rare in the pediatric population. Bleeding associated with pediatric pelvic fractures typically occurs due to solid-organ injuries; therefore, the detection and treatment of these life-threatening injuries should take priority during acute management of children presenting with pelvic fractures.^[17]^ A whole body computer tomograph (CT) scan is recommended in potentially poly-traumatized children because of its rapid availability and high sensitivity, thus providing relevant information to initiate life-saving therapy.^[18]^ Gansslen reviewed the literature on pediatric pelvis fractures, showing that children with pelvic injuries have an average of 5.2 concomitant injuries.^[4]^

In this prospective study we analyzed epidemiological data on children suffering from pelvic fractures. Our study aim was to evaluate any correlation between the severity of pelvic fractures in association with other injured regions and the resulting mortality. We also assessed any correlations between severe pelvic injuries, mild hypotension, and Multiple Slice Computer Tomography (MSCT) findings.

## 2 Methods

### 2.1 TR-DGU

The German Trauma Society's TR-DGU was initiated in 1993.^[19]^ The aim of this multi-center database is the pseudonymized and standardized documentation of severely injured patients. Data are collected prospectively at 4 consecutive time periods from the accident site until hospital discharge: pre-hospital phase, emergency room, intensive care unit (ICU), and discharge. The inclusion criteria are admission to a hospital through the emergency room with subsequent ICU care, or referral to the hospital with critical vital signs and death before admission to the ICU.

The infrastructure for documentation, data management, and data analysis is provided by the Academy for Trauma Surgery- Akademie der Unfallchirurgie GmbH (AUC), a company affiliated with the German Trauma Society. The scientific leadership is provided by the Committee on Emergency Medicine, Intensive Care and Trauma Management (Sektion NIS) of the German Trauma Society. The participating hospitals submit their data pseudonymized to a central database through a web-based application. Scientific data analysis is approved according to a peer-review procedure established by the Sektion NIS.

Most of the participating hospitals are located in Germany (90%), but a rising number of hospitals in other countries have been contributing data as well (these currently include Austria, Belgium, China, Finland, Luxemburg, Slovenia, Switzerland, the Netherlands, and United Arab Emirates). About 35,000 cases from over 600 hospitals are currently being entered into the database per year. Participation in the TR-DGU is voluntary. For certified hospitals associated with the TraumaNetzwerk DGU, however, participation is obligatory for reasons of quality assurance.

The database is the pseudonymized for scientific analyses and guaranteed for both the individual patient and participating hospital.^[20–22]^

The present study is in line with the publication guidelines of the TR-DGU and registered as a TR-DGU project ID 2013-073.

### 2.2 Patients

We analyzed the 2016 database comprising 9684 patients from 2002 to 2015. Inclusion criteria were an Injury Severity Score (ISS) ≥9 and age of 0 to 17 years.

The so-called standard-datasheet was completed by 29.7% (= 2875) of the patients. This datasheet offered more information than the main quality sheet (surgical procedure, fracture classification).

The main focus of this survey was on the pediatric group of patients defined by an age ≤17 years who suffered pelvic fractures.

## 3 Statistical analysis

A descriptive data analysis was performed.

The following variables were used to conduct a descriptive data analysis and a univariate analysis and cross-tabulation.

Statistical significance was defined as *P* *<* .05. Statistics were analyzed using SPSS Version 20.0 (IBM Inc., Armonk, NY).

## 4 Results

A total of 9684 patients ≤17 years were identified in the registry and 1433 children (14.8%) had a severe pelvic injury with an Abbreviated Injury Scale (AIS) pelvis ≥2 (58.3 male and 41.7% female). The mean age was 9.3 years (±4.2) overall, and age-dependent subgroups with an AIS pelvis ≥2 were assessed as follows:

- Group A (1–5 years): 85 of 1284 patients (6.6%)
- Group B (6–10 years): 176 of 1682 patients (10.5%)
- Group C (11–14 years): 308 of 2015 patients (15.3%)
- Group D (15–17 years): 864 of 4703 patients (18.4%).

The number of children with severe pelvic injuries rises with increasing age.

In 75.9% of the cases, children with an AIS pelvis ≥2 were transferred to a level 1 hospital, in 21.4% of the cases to a level 2, and in 3.0% to a level 3 trauma center. We identified no relevant differences in the different age groups analyzed for admission.

Transfer to another hospital after the initial treatment took place in 17.1% to 11.7% of the cases.

Table 1 illustrates each group's characteristics (numbers of children with an AIS pelvis ≥2, mortality, mean ISS, mean stay in the ICU and in the hospital (in days) with standard deviation)

**Table 1:**
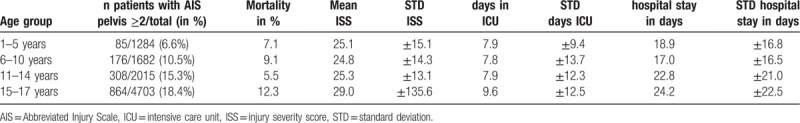
Shows the number of children included in the different age groups with an AIS pelvis ≥2, the mortality, mean ISS, days in ICU, and hospital, each with standard deviation.

**Figure 1:**
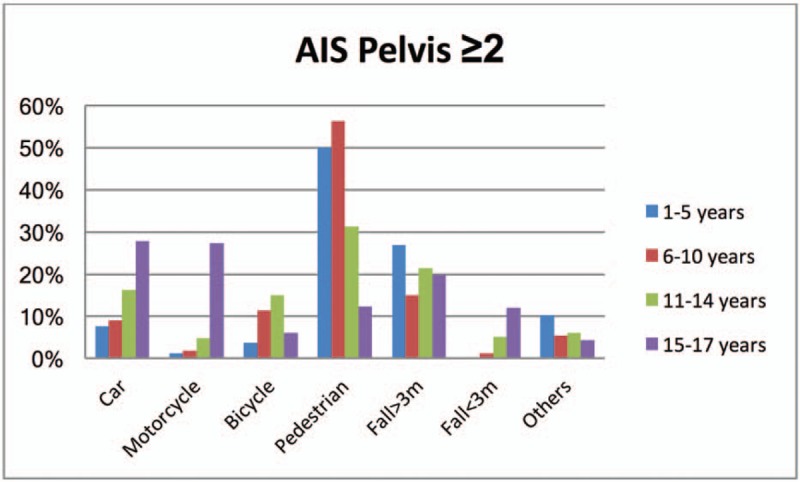
Illustrates the type of accidents of children with a severe pelvic injury (AIS≥2) in the various age groups. An accident as a pedestrian was the most frequent cause of the pelvic injury in young children (1–5 years: 50.0%; 6–10 years: 56.3%; and 11–14 years: 31.1%). Moreover, in the young children up to 5 years, a fall >3m was the cause in 26.9%. AIS = Abbreviated Injury Scale.

Figure 1 illustrates the type of accidents of children with a severe pelvic injury (AIS≥2) in the different age groups. An accident as a pedestrian was the most frequent cause of the pelvic injury in young children (1–5 years: 50.0%; 6–10 years: 56.3%; and 11–14 years: 31.1%). A fall from a height of>3m was the cause in young children up to the age of 5 years in 26.9%.

However, in the analyzed group with severe pelvic fractures (AIS pelvis ≥2)— we noted a overall rate of 58.3% boys and 41.7% girls; in the age-specific subgroups the rate of boys differed from 48.5% to 65.3%.

### 4.1 Mortality

The overall mortality of 9684 pediatric patients was 8.2% in the course of the hospital stay. A lethal outcome was observed in 10.1% of the children presenting an AIS pelvis of ≥2 and in 7.9% of those with an AIS pelvis of <2.

The analysis below includes 8416 patients treated in the hospital where they were first admitted. Data of patients transferred to another hospital during treatment were excluded.

Table 2 summarizes the rate of death in hospital referring to the RISC II score and Standardized Mortality Ratio according to the severity of the pelvic fractures using the Tile A, B, and C classifications.^[23]^ A subgroup of children ≤14 years was analyzed separately. Each group's mortality was lower than the calculated RISC II score.

**Table 2:**
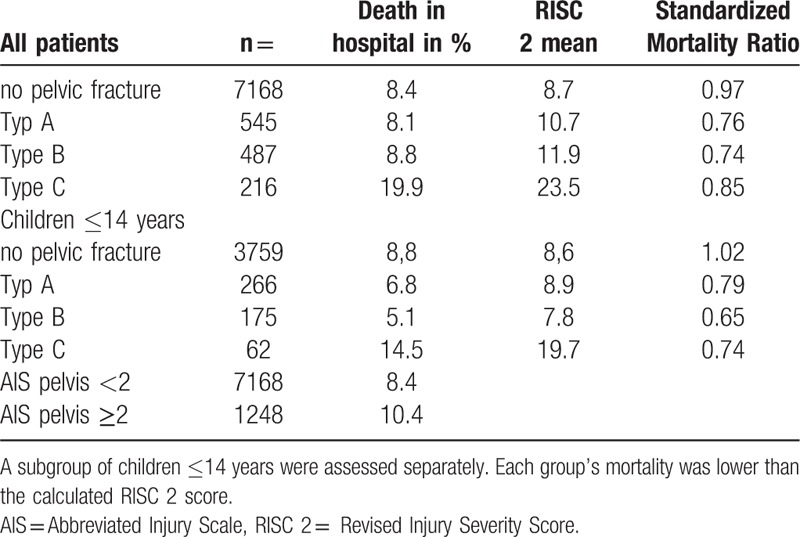
Summarizes the severity of the pelvic fractures using the Tile A, B, and C classification (35) correlating with death in hospital via the RSIC 2 score and Standardized Mortality Ratio.

Table 3 shows the preclinical volume application, number of children with mild hypotension (RR ≤90 mmHG) preclinically or in the ER, and the percentage of CTs and MSCTs done.

**Table 3:**
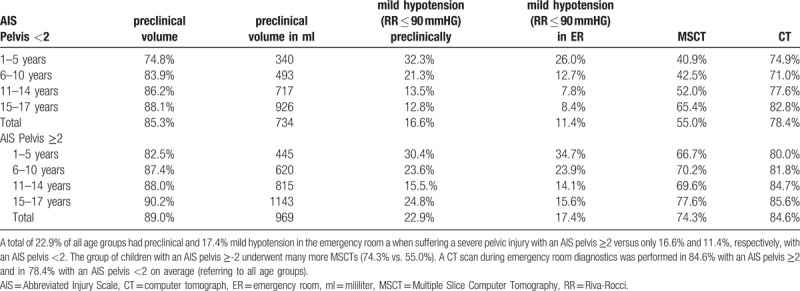
Illustrates the preclinical volume application, the number of children with mild hypotension (RR ≤ 90 mmHG) preclinically or in the ER, and the percentage of CTs and MSCTs.

In the preclinical setting, a total of 22.9%, and in the ER a total of 17.4% of the children of all age groups had mild hypotension when suffering a severe pelvic injury with an AIS pelvis of ≥2—compared to only 16.6% (preclinical setting) and 11.4% (ER) respectively in children with an AIS pelvis of <2. The rate of MSCTs was significantly higher in the group of children with an AIS pelvis of ≥2 (74.3% vs. 55.0%). The rate of CT scans performed during emergency room diagnostics was 84.6% in the patients with an AIS pelvis of ≥2 and 78.4% in those with an AIS pelvis of <2 (Table 5).

**Table 5:**
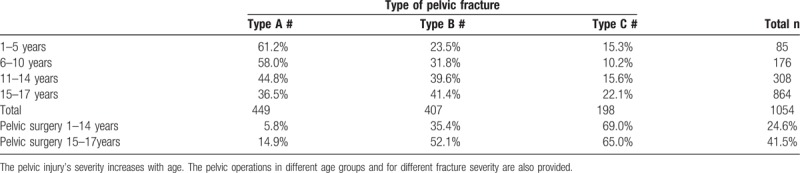
Shows the Tile classification analysis in the different age groups of all patients with pelvic injuries.

The group of children with severe pelvic injuries suffered a higher incidence of severe thorax and abdominal injuries (AIS ≥ 2) (Table 4). Details are displayed below.

**Table 4:**
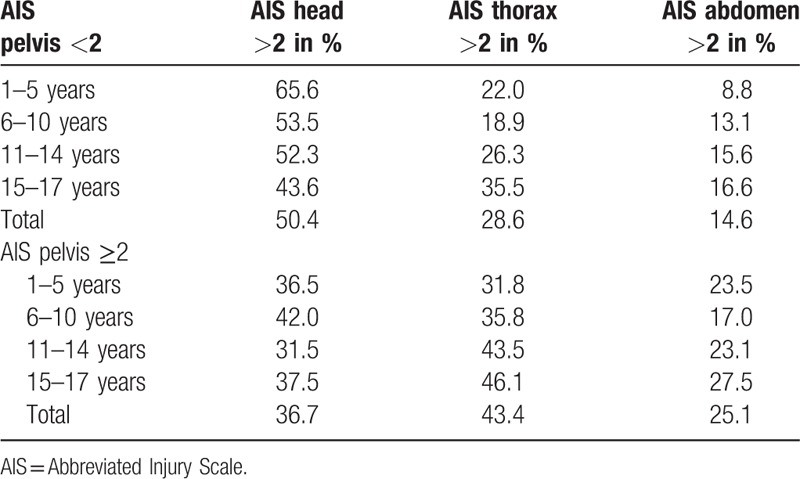
Shows that those children with severe pelvic injuries had a higher incidence of severe thorax and abdominal injuries in all age groups (AIS≥- 2).

Fracture severity in the different age groups according to the Tile classification. The severity of the pelvic injuries rises with age. The rate of pelvic surgery performed in patients ≤14 years and >14 years according to the fracture severity is presented.

Table 6 shows the percentage of surgery the different age groups underwent in and the pelvic injury's severity. The rate of subsequent surgical interventions increases in conjunction with a rise in the AIS pelvis: AIS 2: 7.6%, AIS 3: 35.0%, AIS 4: 65.6%, AIS 5: 61.5%.

**Table 6:**
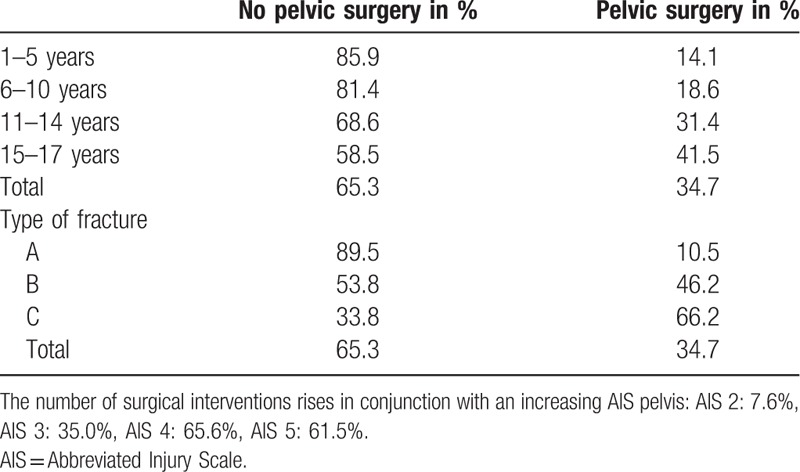
Shows the percentage of surgery performed in correlation with the given age group and severity of the pelvic injury.

## 5 Discussion

In line to the literature, we noted that 72.3% of all pelvic fractures with AIS ≥2 in children were caused by traffic accidents.^[9,24]^ By Analyzing the register's data has enabled us to describe relevant parameters in the treatment of pelvic fractures in an extraordinarily large cohort of children.

Regarding the patients’ characteristics: the findings of Gänsslen et al (who analyzed different studies on pediatric pelvic fractures) were in line with this study's observations. They analyzed a mean patient age of 9 years and a mean ISS of 15.7 points, as well as male predominance with a male/female ratio of approximately 1.4:1.^[12]^ Moreover Gänsslen et al cite a 60% to 80% rate of type A injuries, 10% to 35% type B injuries, and 10% to 16% type C injuries.

In our analysis, we separated children up to the age of 14 years with the knowledge that the epiphyseal plate in the acetabulum closes between 14 and 16 years.^[25]^ Gansslen et al summarized in 10 studies on pediatric pelvic fractures a mean age of 9 years, similar to our mean age of 9.3 years.^[12]^ To provide more detailed age-stratified information, we assessed 4 different age groups of children with pelvic fractures in this study.

Multiple approaches for the operative treatment of pelvic fractures in children have been published with different rates of surgical interventions from 0.6% to 30% following a pelvic fracture and reporting comparable rates of external and internal fixation.^[12]^ According to our registry, 24.6% of the children aged ≤14 years underwent a surgical intervention, whereas 41.6% of the children and adolescents aged 15 to 17 years did. We noted a strong association between operative treatment and the severity of the pelvic injury, for example, 65% to 69% of the patients with a Tile C fracture underwent surgery.

There is ample evidence that conservatively treated; displaced pelvic fractures in children can lead to pelvic asymmetry and poor clinical outcomes. Thus, many authors have focused on surgically stabilizing the pelvic ring.^[15,26,27]^ The standard indications for the operative fixation of pelvic fractures are:

- open fractures
- additional hemorrhage control during resuscitation^[28]^
- optimization of patient mobility
- prevention of deformity in severely displaced fractures^[8,29]^
- enhancement of patient mobility in particular situations (e.g., polytrauma).

With this knowledge, it becomes clear that only displaced fractures require surgical reduction and stabilization,^[15,28,29]^ and only case descriptions are reported in the literature.^[13]^ Unfortunately, we had no additional information to clarify why only 66.2% of Tile C fractures were treated surgically. Pelvic fracture patterns in children typically differ from those seen in adults. The juvenile pelvis is more elastic, and has a thicker layer of cartilage and more flexibility in the symphysis and sacroiliac (SI) joints due to their specific anatomic characteristics.^[30]^ Pelvic fractures therefore occur as a result of a high-energy trauma and are often associated with polytrauma.^[31,32]^ An analysis from the American National Inpatient Pediatric Database revealed that children with pelvic injuries presented 5.2 concomitant injuries on average.^[33]^

A summary of the latest literature shows that 83.3% of all pediatric pelvic injuries were due to high-energy trauma. The US analysis also reveals that a pedestrian being struck by a car was the mechanism in 57.8%, a motor vehicle passenger was injured in 17.8%, a bicyclist in 4.9%, and a motorcyclist in 0.6%. A fall from a height was responsible for causing a pediatric pelvic fracture in 9.2%. Crush injuries (2.2%), injuries sustained during sport activities (2.1%), and farm accidents (0.5%) were uncommon.^[12]^ A key prognostic injury mechanism is the history of roll-over or crush (ISS up to 40 points, 86.6%. associated injuries, 20% mortality rate >70% local complication rate).^[34]^ Although polytraumatized children should undergo CT scans,^[35]^ we identified an overall rate of CT scans in only 84.6% of the children with relevant pelvic injuries in this patient collective.

Different emergency devices are available to for the stabilization of an instable pelvis. Especially important for the immediate treatment of pelvic fractures in children a pelvic slings, pelvic bed sheets, or a pelvic binder.^[36]^ Antishock trousers are no longer recommended in adults because of the high rate of complications^[37]^ and the authors do not advise them for use in children. As our registry findings also reveal, stabilizing through external fixation is the most common stabilization technique for pediatric pelvic fractures.^[26,27,37]^ McIntyre et al analyzed a rate of 60% of controlled bleeding after external fixation.^[38]^ The address the instability of the posterior pelvic ring the pelvic C-clamp is an adequate option.^[39]^

Only when the child is in a stable condition a definitive reduction and internal fixation with symphyseal plating, anterior plating of the SI-joint, or/and application of transiliosacral screws is recommended.^[40]^ The angiography or embolization and pelvic packing are useful techniques to control pelvic hemorrhage. Angiography and embolization to stabilize hemodynamics in pediatric patients with pelvis fractures can succeed, but reported time intervals between admission and the start of embolization range from 12 to 15 hours in an international study, and only 62 minutes in a German study.^[41,42]^ External fixation was the most often applied method in children and adults, however, the advantage at a younger age is that it is more frequently administered as definitive care. In general, children rarely seem to suffer from any thrombosis/thrombembolism, acute respiratory distress syndrome (ARDS), multiorgan failure (MOF), or neurologic deficit, nor any septic MOF, even in cases of pelvic fractures.^[43]^

To summarize: external fixation seems to be an appropriate and minimally-invasive treatment for most instable pelvic fractures in children. Nevertheless, for initial treatment, binding an unstable pelvic fracture (i.e., in a preclinical or ER context) and angioembolization during the first clinically-stable hours should be considered as treatment options for children.

The fact that this study is an evaluation of a prospective multicenter registry represents both its strength and a limitation. On the 1 hand, including patients from several institutions best reflects a country's therapeutic reality. On the other hand, the authors must rely on different kinds of trauma centers (of different levels) contributing to the registry, thus treatment protocols depend on each institution's environment and routine.

## 6 Conclusion

A very high number of children with pelvic fractures could be analyzed in this register study in terms of fracture severity, mortality, treatments performed, and other relevant factors. A pediatric Tile C fracture is associated with the highest mortality rate of 14.5% in the subgroup of children ≤14 years, especially in those with pelvic fractures of an AIS ≥2.

Trauma surgeons involved in pediatric trauma care should have knowledge of the age-specific anatomy in children and adolescents. Great care must be taken when examining patients who may have suffered relevant instable pelvic fractures requiring surgical intervention and stabilization.

## Author contributions

**Conceptualization:** Jörn Zwingmann, Lisa Hohloch, Helge Eberbach, Peter C Strohm.

**Data curation:** Jörn Zwingmann, Rolf Lefering, Thorsten Hammer.

**Formal analysis:** Jörn Zwingmann, Rolf Lefering, Helge Eberbach, Thorsten Hammer.

**Methodology:** Jörn Zwingmann, Rolf Lefering, Dirk Maier, Lisa Hohloch, Mirjam Neumann, Thorsten Hammer.

**Project administration:** Jörn Zwingmann.

**Supervision:** Norbert P Südkamp, Thorsten Hammer.

**Validation:** Jörn Zwingmann, Thorsten Hammer.

**Visualization:** Helge Eberbach, Mirjam Neumann.

**Writing – original draft:** Jörn Zwingmann.

**Writing – review & editing:** Jörn Zwingmann, Dirk Maier, Peter C Strohm.

Jörn Zwingmann orcid: 0000-0001-8755-6383
